# Risk of a major adverse cardiovascular event (MACE) following first-ever hospitalisation for acute gout: a Western Australian population-level linked data study.

**DOI:** 10.23889/ijpds.v7i3.1805

**Published:** 2022-08-25

**Authors:** Derrick Lopez, David Preen, Warren Raymond, Charles Inderjeeth, Kevin Murray, Hans Nossent, Girish Dwivedi, Helen Keen

**Affiliations:** 1 Karolinska Institute

## Objectives

Cardiovascular disease is the largest contributor of increased mortality in patients with gout. Acute inflammation as seen with gout attacks may have a mechanistic role in developing Major Adverse Cardiovascular Events (MACE). We examined the temporal relationship between admission to hospital with acute gout and MACE.

## Approach

Linked inpatient and mortality data from the Western Australian Rheumatic Disease Epidemiology Registry were used. We identified patients with an incident acute gout (index) hospitalisation and admission or death records due to MACE (composite of acute coronary syndrome, stroke, heart failure, cardiovascular death). The risk of MACE during the index post-discharge period (1-30 days after index admission) and control period (365 days prior to index admission and 365 days post-discharge) was determined using a self-controlled case-series (SCCS) design. Conditional fixed-effects Poisson regression was used to obtain incidence rate ratios (IRR). Sensitivity analyses were performed excluding deaths and 180-day events.

## Results

We identified 962 patients (mean age=76.2 years [SD=12.2]; 66.8% male) with incident acute gout admission and documented MACE during the control and/or index post-discharge periods. 917 (95.3%) patients experienced MACE during the control period and 114 (11.9%) during the index post-discharge period. The rate of MACE during the control and post-discharge periods were 0.84 and 1.44 events per person-year, respectively, with an IRR=1.67 (95% CI: 1.38-2.02) for the post-discharge period compared with the control period from regression analysis. Sensitivity analyses excluding deaths and 180-day events were IRR=1.68 (95% CI=1.29-2.20) and IRR=1.66 (95% CI=1.34-2.07) respectively.

## Conclusion

Our self-controlled case-series study using linked administrative data found an increased risk of MACE during the 30 days after discharge for index gout hospitalisation. This suggests a temporal association between acute inflammation and MACE.

